# Model-based geostatistics enables more precise estimates of neglected tropical-disease prevalence in elimination settings: mapping trachoma prevalence in Ethiopia

**DOI:** 10.1093/ije/dyab227

**Published:** 2021-11-13

**Authors:** Benjamin Amoah, Claudio Fronterre, Olatunji Johnson, Michael Dejene, Fikre Seife, Nebiyu Negussu, Ana Bakhtiari, Emma M Harding-Esch, Emanuele Giorgi, Anthony W Solomon, Peter J Diggle

**Affiliations:** Centre for Health Informatics, Computing and Statistics, Lancaster Medical School, Lancaster University, Bailrigg, Lancaster, UK; Centre for Health Informatics, Computing and Statistics, Lancaster Medical School, Lancaster University, Bailrigg, Lancaster, UK; Centre for Health Informatics, Computing and Statistics, Lancaster Medical School, Lancaster University, Bailrigg, Lancaster, UK; Michael Dejene Public Health Consultancy Services, Addis Ababa, Ethiopia; Disease Prevention and Control Directorate, Federal Ministry of Health, Addis Ababa, Ethiopia; Children’s Investment Fund Foundation, London, UK; International Trachoma Initiative, Task Force for Global Health, Decatur, GA, USA; Department of Clinical Research, Faculty of Infectious and Tropical Diseases, London School of Hygiene & Tropical Medicine, London, UK; Centre for Health Informatics, Computing and Statistics, Lancaster Medical School, Lancaster University, Bailrigg, Lancaster, UK; Department of Control of Neglected Tropical Diseases, World Health Organization, Geneva, Switzerland; Centre for Health Informatics, Computing and Statistics, Lancaster Medical School, Lancaster University, Bailrigg, Lancaster, UK

**Keywords:** Neglected tropical diseases, trachoma prevalence, elimination, precision, geostatistics, exceedance probabilities

## Abstract

**Background:**

As the prevalences of neglected tropical diseases reduce to low levels in some countries, policymakers require precise disease estimates to decide whether the set public health targets have been met. At low prevalence levels, traditional statistical methods produce imprecise estimates. More modern geospatial statistical methods can deliver the required level of precision for accurate decision-making.

**Methods:**

Using spatially referenced data from 3567 cluster locations in Ethiopia in the years 2017, 2018 and 2019, we developed a geostatistical model to estimate the prevalence of trachomatous trichiasis and to calculate the probability that the trachomatous trichiasis component of the elimination of trachoma as a public health problem has already been achieved for each of 482 evaluation units. We also compared the precision of traditional and geostatistical approaches by the ratios of the lengths of their 95% predictive intervals.

**Results:**

The elimination threshold of trachomatous trichiasis (prevalence ≤ 0.2% in individuals aged ≥15 years) is met with a probability of 0.9 or more in 8 out of the 482 evaluation units assessed, and with a probability of ≤0.1 in 469 evaluation units. For the remaining five evaluation units, the probability of elimination is between 0.45 and 0.65. Prevalence estimates were, on average, 10 times more precise than estimates obtained using the traditional approach.

**Conclusions:**

By accounting for and exploiting spatial correlation in the prevalence data, we achieved remarkably improved precision of prevalence estimates compared with the traditional approach. The geostatistical approach also delivers predictions for unsampled evaluation units that are geographically close to sampled evaluation units.

Key Messages

- At the very low prevalence required to meet thresholds defining *elimination as a public health problem* for some neglected tropical diseases, traditional statistical methods of estimating prevalence yield imprecise estimates.
- Model-based geostatistics (MBG) borrows strength of information across different sampled locations to an extent determined by the strength of the estimated spatial correlation between locations.
- In our study, the MBG approach delivered trachomatous trichiasis prevalence estimates that were, on average, 10 times more precise than those given by the traditional approach to estimating trachomatous trichiasis prevalence; allowed assessment of elimination status to be made with greatly reduced uncertainty for a given sample size; and enabled estimation of disease prevalence in unsampled evaluations units sufficiently close to sampled locations.
- If cost considerations are paramount, MBG allows elimination surveys to be designed with substantially fewer sampled locations than the traditional approach while achieving the same, or better, precision.

## Introduction

Neglected tropical diseases rank among the world’s greatest global health problems because of their substantial contribution to global morbidity, disability and mortality.^1–3^ The highest burdens of neglected tropical diseases occur in the tropical and subtropical regions of the world, affecting the world’s poorest people and exacerbating poverty through their detrimental effects on work productivity, child development and women’s health.^1^^,^^4^ In the London Declaration, 22 partners including endemic countries, non-governmental organizations, pharmaceutical companies and donors committed to controlling, eliminating or eradicating at least 10 neglected tropical diseases by the year 2020.^5^ Experts in neglected tropical diseases are generally optimistic about the prospects of elimination for lymphatic filariasis, onchocerciasis and trachoma,^6^ although the dates by which success could be anticipated have recently been revised.^7^

For selected neglected tropical diseases, *elimination as a public health problem* is defined as the reduction of prevalence in a given geographic area to a disease-specific level set by the World Health Organization (WHO).^8^ Determining the elimination status of an at-risk population is of critical importance, but complete ascertainment of disease or reinfection status for every member of an at-risk population is impractical. Therefore, it is imperative that prevalence surveys are designed, and the resulting data analysed, using the most efficient statistical methods. In this study, we used extensive prevalence survey data on trachoma in Ethiopia to demonstrate how the application of model-based geostatistical analysis can achieve very substantial gains in precision over the traditional statistical analysis methods that are currently used in this context.

Trachoma is the leading infectious cause of blindness globally.^9^^,^^10^ The disease is caused by repeated infection of the conjunctiva with particular strains of the bacterium *Chlamydia trachomatis*.^10^ Higher forces of infection are associated with poor facial cleanliness, overcrowding, absence of functional latrines and overall poor community-level sanitation.^11–15^ Infection often results in conjunctival inflammation with follicles (‘active trachoma’) that may meet the WHO’s definition of the sign ‘trachomatous inflammation–follicular’ (TF).^16^ TF prevalence is highest in young children, who also harbour the highest bacterial loads.^14^^,^^17–20^ In individuals with repeated reinfections, scarring of the conjunctivae with consequent in-turning of the eyelashes can occur. This is called trachomatous trichiasis.^21–26^ The in-turned eyelashes cause pain at each blink and may, over time, cause scarring of the cornea, leading to visual impairment and blindness.^16^^,^^27^

Elimination of trachoma as a public health problem is defined as (i) a prevalence of trachomatous trichiasis unknown to the health system of <0.002 (0.2%) in adults aged ≥15 years and (ii) a prevalence of TF <0.05 (5%) in children aged 1–9 years, in each formerly endemic evaluation unit; plus (iii) the presence of a system to identify and manage incident cases of trachomatous trichiasis, which are expected to arise for many years after the prevalence thresholds (i) and (ii) are met. An evaluation unit for assessing elimination is defined as the normal administrative unit for healthcare management, which typically contains a population of between 100 000 and 250 000 people.^28–31^

To achieve these endpoints, the WHO Alliance for the Global Elimination of Trachoma by 2020 recommends use of the SAFE strategy of Surgery to correct trachomatous trichiasis, antibiotics to clear *C. trachomatis* infection, and facial cleanliness and environmental improvement to reduce transmission. Ethiopia has been the country with the highest levels of trachoma prevalence^32^^,^^33^ and has implemented the SAFE strategy to push towards trachoma elimination.

Several factors have impeded the prospects of trachoma elimination. First, it has been challenging to control the recrudescence of infection after mass drug administration with azithromycin, the preferred antibiotic for active trachoma treatment. Reinfection has occurred repeatedly even under high mass drug-administration coverage.^34^ The outbreak of the COVID-19 pandemic and the subsequent suspension of some control activities are likely to facilitate a faster resurgence. Second, there has been a high recurrence rate of trichiasis after surgical correction.^35^ Third, even after no infections can be detected at the population level, inflammation in children’s conjunctivae may continue to occur.^36^ Fourth, the relationship between environmental factors and trachoma are poorly understood and, in some areas, environmental corrections have failed to yield the expected impact.^37^ More research in these areas is needed to better understand how the SAFE strategy could be optimized to enhance the chances of trachoma elimination.

A critical challenge in trachoma elimination is establishing the prevalence of the disease in elimination settings, and hence determining whether or not elimination has been achieved. At the very low prevalence levels required to meet elimination thresholds, prevalence estimates are typically very imprecise. Two sources of imprecision exist. First, estimates are sure to be imprecise if the entire population at risk is not tested using the perfect diagnostic tool in a perfect manner. Second, imprecision arises from the sampling design used to collect the data, and the statistical methods and models used to analyse them. As prevalence decreases and the disease becomes rare, increasingly large sample sizes are needed to estimate the prevalence of the disease to a high level of precision, which is needed to increase the chances of making the correct decision on whether or not elimination has been achieved and avoiding inconclusive results.

The 3rd Global Scientific Meeting on Trachoma, in an attempt to solve the problem of imprecise estimates, stipulated the use of population-based prevalence surveys through cluster random sampling powered to detect a TF prevalence of 4% with the absolute precision of ±2% at the evaluation-unit level, which would require an estimated 970 children aged 1–9 years per survey.

Meanwhile, to estimate with 95% confidence an expected trachomatous trichiasis prevalence of 0.2% with an absolute precision of ±0.2% would require an estimated 2818 adults aged ≥15 years or data from 30 first-stage clusters. However, the challenge is that many impact and surveillance surveys are primarily designed to estimate the prevalence of TF in 1– to 9-year-olds and result in <30 first-stage clusters and <2818 adults aged ≥15 years.^38^ Against this background, we applied geospatial methods to improve the precision of trachomatous trichiasis prevalence estimates using data from impact and surveillance surveys conducted in Ethiopia, despite their apparent lack of power.

Environmental and some socio-economic factors are correlated in space, and so are the diseases that depend on them. When a disease is spatially correlated, its prevalence at a geographical location informs the prevalence at other locations, with the strength of the correlation increasing with decreasing distance apart. The current method of estimating trachomatous trichiasis prevalence does not include spatial correlation in estimating the risk of infection within or between evaluation units, thus implicitly assuming that data from different geographical locations are independent. However, when substantial spatial correlation exists in the data, accounting for it and exploiting it in a model-based geostatistics (MBG) framework have several advantages. First, inferences on estimated regression relationships are more reliable because the data analysis accounts for additional unexplained variability in the data. Second, there are substantial improvements in the precision of prevalence estimates because the information provided by the spatial correlation reduces the uncertainty in the data. Third, the estimated spatial correlation allows predictions at unsampled geolocations that are close enough to sampled geolocations. The aim of this paper was to apply MBG to impact and surveillance survey data from Ethiopia to determine whether this approach enables more precise trachomatous trichiasis prevalence estimates to be obtained.

## Methods

### Sampling and data

To determine whether the elimination prevalence target for trachomatous trichiasis has been reached in formerly endemic evaluation units, acceptable approaches are: (i) population-based prevalence surveys powered at the evaluation-unit level (i.e. ‘the normal administrative unit for healthcare management, consisting of a population unit of 100 000–250 000 persons’); (ii) house-to-house case searches (which could be integrated with other public health activities); or (iii) a combination of data from multiple adjacent evaluation units.^39^

We used existing data from trachoma impact and surveillance surveys powered at the evaluation-unit level, conducted in 2017, 2018 and 2019 in the following Ethiopian regions: Tigray; Oromia; Amhara; Gambella; Benishangul-Gumuz; Southern Nations, Nationalities, and Peoples’ Region. All the surveys were conducted with Tropical Data support, which uses the same standardized, robust methodology as used in the Global Trachoma Mapping Project.^40^

Impact and surveillance surveys were conducted according to the schedule recommended by the WHO.^41^ Impact surveys were carried out in evaluation units ≥6 months after completion of the WHO-recommended numbers of rounds of annual antibiotic mass drug administration. Surveillance surveys were carried out in evaluation units that had achieved the WHO TF elimination threshold of 0.05 among children aged 1–9 years, measured at impact surveys, and had then stopped mass antibiotic administration for ≥2 years before being resurveyed.

The design for both an impact and a surveillance survey conformed with the WHO recommendations^38^ and included the following basic elements. First, in each evaluation unit, 20–30 villages were selected using a probability-proportional-to-population-size method. Second, within each selected village, a group of 30 households was selected using compact segment sampling. Finally, every resident aged ≥1 year in each selected household was invited to participate.^30^^,^^38^^,^^42^ Both eyes of consenting residents were examined for trachomatous trichiasis and TF using the WHO’s simplified grading system for trachoma.^16^ For our analysis, we considered a case of trachomatous trichiasis to be anyone aged ≥15 years with at least one eyelash from the upper or lower eyelid touching the eyeball, or evidence of recent epilation of in-turned eyelashes from the upper or lower eyelid.

### Estimating evaluation-unit-level prevalence

The prevalence of trachomatous trichiasis is higher in females and increases with age.^43–45^ However, daytime population-based surveys are likely to over-represent older individuals and females. The data analysis therefore needs to adjust for age and gender so that predictions correctly reflect the demography of the population. We binned age in 5-year bands from 15 to 69 years and a single band for ≥70 years.^46^

### Traditional approach

In our implementation of the traditional approach to estimation of population-based trachomatous trichiasis prevalence from cluster-level proportions, we adjusted for age and gender using the 2007 Ethiopia census data as the standard population. We then developed 95% confidence intervals by bootstrapping adjusted cluster-level proportions over 10 000 replicates. For districts with no sampled cases, we estimated upper confidence interval limits as one-sided 97.5% exact binomial confidence intervals.

### Geostatistical approach

Our geostatistical model was a logistic regression with the addition of two kinds of random effects: spatially correlated residual variation, which we modelled using a stationary Gaussian process; and spatially uncorrelated residual variation, which we modelled as Gaussian noise.

A symbolic description of the model is

(1) $$\text{log}\;\;\text{odds}\;\text{of}\;\text{trachomatous}\;\text{trichiasisin}\;\text{individual}=\alpha_{t}+\beta_{k}+\text{spatially correlated residual variation}+\text{spatially}\;\text{uncorrelated residual variation,}$$

where *α_t_* is the regression parameter for the survey year (2017, 2018 or 2019) and *β_k_* is the regression parameter for the age–gender class of the individual. Informally, the right-hand side of Equation (1) divides the variation in the log-odds of prevalence into an explained component (here, year of survey and age–gender class) and an unexplained component (here, partitioned into spatially correlated and spatially uncorrelated subcomponents, in proportions to be determined by the data). Technical details of the model and model building process are given in the Supplementary Appendix (available as Supplementary data at *IJE* online).

To estimate local trachomatous trichiasis prevalence, we placed a 5 × 5 km grid on the study region. Within each grid-cell, we then estimated trachomatous trichiasis prevalence for each age–gender class and computed the class-weighted prevalence, where the weights were the proportions of the population in each class according to the 2007 census of Ethiopia.

Our geostatistical evaluation-unit-level predictions needed to account for the within-evaluation-unit heterogeneity in population density. We used the class-weighted trachomatous trichiasis prevalence from all grid-cells within an evaluation unit to estimate evaluation-unit-wide prevalence as a population-weighted prevalence, incorporating estimated population counts from WorldPop.^47^

We obtained 10 000 predictive samples for each evaluation unit and from these computed the following quantities: the *evaluation-unit-wide prevalence* was the mean of the predictive samples of prevalence for that evaluation unit; the associated *95% prediction interval* was the range from the 2.5th to 97.5th percentiles; the *probability of elimination* was the proportion of predictive samples less than the elimination threshold.

The Supplementary Appendix (available as Supplementary data at *IJE* online) gives technical details of the modelling, model-fitting, prediction algorithm and model-validation.

## Results

### Sampling and data

**Figure 1:**
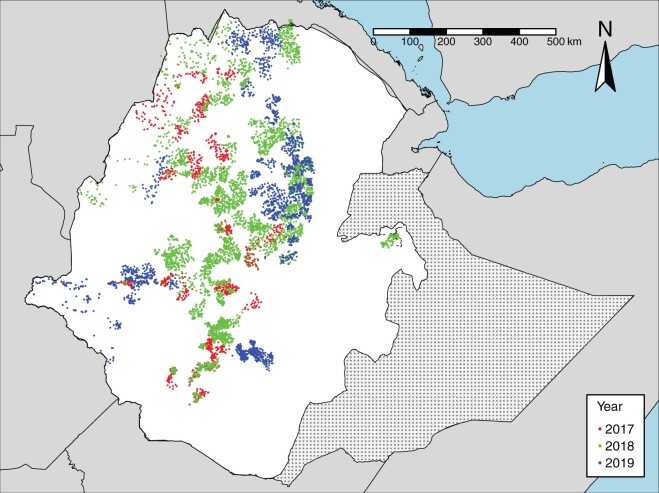
Cluster locations colour-coded according to their year of survey

We collected data from 458 678 individuals (268 297 females and 190 381 males) sampled from 7447 clusters. Figure 1 shows the locations of all clusters, colour-coded according to their empirical prevalences. The overall empirical prevalence was 0.021 with interquartile range 0.000–0.032.

### Model estimates

**Figure 2:**
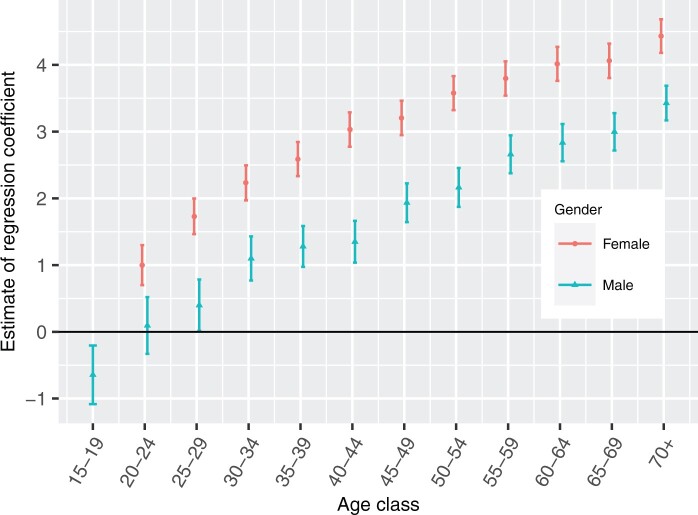
Point estimates of the log-odds of prevalence in the age–gender classes and associated 95% confidence intervals

The estimates of the log-odds of trachomatous trichiasis prevalence in the age–gender classes (Figure 2) show, as expected,^14^^,^^24^ that trachomatous trichiasis prevalence is higher in females and increases with age. The higher trachomatous trichiasis prevalence in women has been attributed to their propensity to spend more time than men caring for children,^48^ who constitute the highest prevalence group and harbour the highest parasite loads.

**Figure 3:**
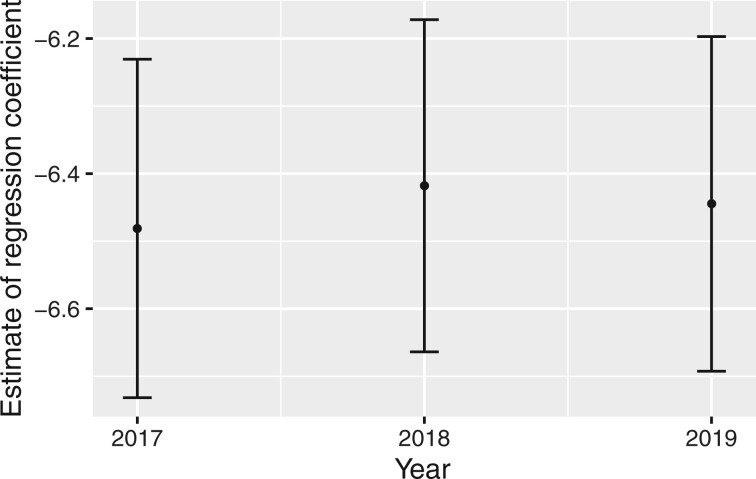
Point estimates of the log-odds of prevalence in different years and associated 95% confidence intervals

The estimates of the log-odds of trachomatous trichiasis prevalence in different years (Figure 3) show subtle differences and the three 95% confidence intervals largely overlap.

**Figure 4:**
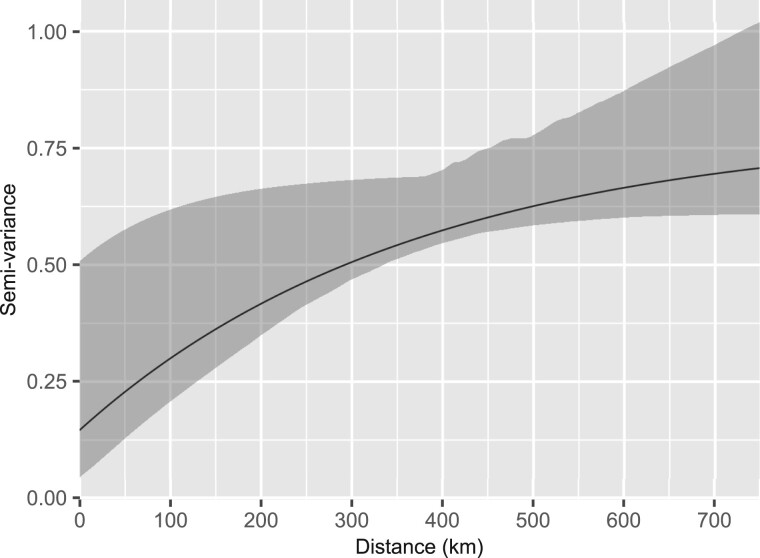
Estimates of the residual spatial correlation structure (solid line) and its 95% confidence region (grey area)

Figure 4 shows the structure of the estimated residual spatial correlation in the form of a variogram. A rising trend indicates the presence of spatial variation.

### Prediction of prevalence and probability of elimination

**Figure 5:**
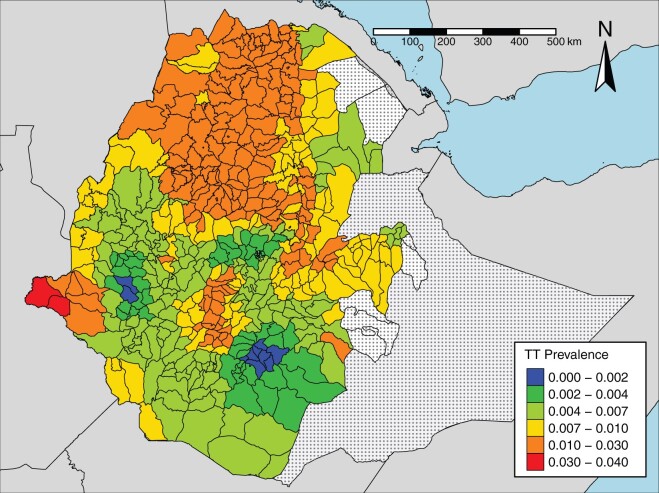
Map of the predicted prevalence for the evaluation units

Figure 5 shows the predicted trachomatous trichiasis prevalences at the evaluation-unit level. Predicted prevalences were less than the elimination threshold of 0.002 in nine evaluation units. These evaluation units and their respective predicted prevalences are: Ale, 0.0019; Arbe Gona, 0.0013; Aroresa, 0.0016; Bensa, 0.0014; Bore, 0.0017; Dara, 0.0018; Hulla, 0.0013; Kokosa, 0.0018; and Metu, 0.0019. Summary statistics of the distribution of predicted prevalences over all evaluation units are: Minimum = 0.00130; 1st Quartile = 0.0030; Median = 0.0077; Mean = 0.0090; 3rd Quartile = 0.0114; Maximum = 0.0353.

**Figure 6:**
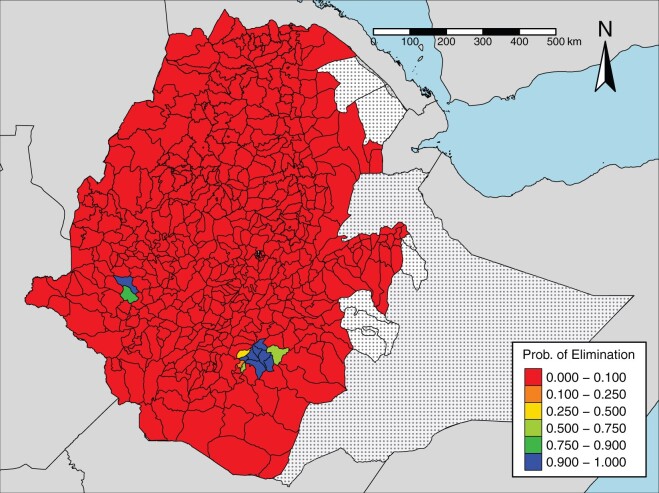
Map of the probability of elimination, given an elimination threshold of 0.002 of TT

Figure 6 shows the probability, for each evaluation unit, that trachomatous trichiasis prevalence is below the elimination threshold of 0.002. This probability is close to zero in most evaluation units. Eight of the 482 evaluation units met the elimination criteria, with a probability of ≥0.9. These evaluation units are those with a predicted prevalence of <0.002, with the exception of Ale. Conversely, with a probability of ≥0.9, 469 evaluation units have *not* achieved the elimination threshold. One evaluation unit (Wenago) has achieved elimination with a probability of 0.88. For all remaining evaluation units, the probability of elimination lies between 0.45 and 0.65.

Detailed predictions (prevalence, 95% confidence interval and probability of elimination) for all 482 evaluation units are given in Supplementary Table S1 (available as Supplementary data at *IJE* online).

### Comparison of the traditional approach with the geostatistical approach

For each of the 257 evaluation units that contain sampled clusters, we used the traditional approach to estimate prevalence and its associated 95% confidence interval. We then compared these estimates with those of the geostatistical approach by calculating the difference between the traditional and geostatistical prevalence estimates and the ratio of the length of the traditional 95% confidence interval to the 95% geostatistical prediction interval. The *differences* between prevalences across the 257 evaluation units can be described by the following summary statistics: Minimum = 0.0000; 1st Quartile = 0.0018; Median = 0.0048; Mean = 0.0066; 3rd Quartile = 0.0086; Maximum = 0.1048. Judged relative to the elimination threshold of 0.002, these are not negligible differences. Summary statistics for the ratio of the traditional to the geostatistical 95% intervals are: Minimum = 3.5; 1st Quartile = 7.9; Median = 9.9; Mean = 17.2; 3rd Quartile = 14.9; Maximum = 146.7. This represents a dramatic improvement in efficiency. Results for each evaluation unit are given in Supplementary Table S2 (available as Supplementary data at *IJE* online).

We did not find appreciable differences in the prevalence of trachomatous trichiasis over the years 2017, 2018 and 2019. This suggests that, notwithstanding progress to date, Ethiopia may need more intensive interventions to achieve nationwide elimination.

## Discussion

We mapped the evaluation-unit-level prevalence of trachomatous trichiasis in Ethiopia and the probability that the prevalence is less than the elimination threshold of 0.002 (0.2%). We then illustrated the gains in precision of prevalence estimates achieved by applying geostatistical methods of estimation over the traditional approach by comparing the respective lengths of the 95% predictive intervals of evaluation-unit-level prevalence. Through Monte Carlo methods, we showed that there is a considerable spatial correlation in trachomatous trichiasis risk in Ethiopia and that exploiting this information in an MBG framework leads to substantial improvements in the precision of trachomatous trichiasis prevalence estimates at the evaluation-unit level.

In November 2018, the 4th Global Scientific Meeting on Trachoma agreed that national programmes could use a combination of data from multiple adjacent evaluation units to assess whether the elimination prevalence target for trachomatous trichiasis has been reached.^39^ This presupposes that trachomatous trichiasis prevalence in an evaluation unit can be, to some extent, informed by trachomatous trichiasis prevalence in adjacent evaluation units, or that decision-makers would accept trachomatous trichiasis prevalence estimates calculated for larger population units. Estimates based on the pooling of data over multiple evaluation units leads to reduced variance, but at the risk of introducing bias. This immediately raises the question: Over what geographical scale can data legitimately be pooled?^39^ One strength of the geostatistical approach is that it allows the data to answer the question rather than imposing an arbitrary, predefined rule. Our model-based geostatistical framework assumes that, after adjusting for relevant covariates, the residual component of variation in prevalence is spatially correlated, but allows the data to determine the strength and spatial scale of this phenomenon.

The current conventional approach to assess whether the trachomatous trichiasis elimination threshold has been met is through calculating a point estimate of prevalence with an attached confidence interval. We have shown through the Ethiopian trachomatous trichiasis case study that the resulting confidence intervals can be an order of magnitude wider than the corresponding geostatistical prediction intervals. This dramatic improvement in efficiency from using a geostatistical approach stems from two quite different considerations. First, the geostatistical approach in effect chooses the optimal pooling of data from spatially neighbouring locations to construct a spatially continuous surface of predicted prevalences that can then be aggregated to whatever set of geographical units is relevant for public health decision-making in context. Second, *it answers the right question*. The confidence intervals used in the conventional approach measure the uncertainty with which prevalence can be estimated over a hypothetical universe of repeated realizations of the disease process. Geostatistical prediction intervals measure the uncertainty for the particular realization of the disease process that has actually occurred.

Another advantage of applying MBG in this context is that it allowed us to quantify uncertainty by using exceedance probabilities, which we argue directly answer the policymakers’ question of whether or not elimination has been achieved, rather than relying on point estimates and confidence intervals that are difficult to interpret in relation to the policy-relevant question and consequently the chances of right/wrong decisions. Importantly, it reports for each evaluation unit the *probability* that elimination has been achieved, thereby forcing consideration of the uncertainty that is necessarily attached to any declaration that elimination has or has not been achieved.

Impact and surveillance surveys do not have the primary power to estimate trachomatous trichiasis prevalence using the traditional approach unless they meet the requirement of trachomatous trichiasis-only surveys, namely a sample size of 2818 adults or 30 clusters within an evaluation unit.^49^ A strength of the geostatistical analysis is that more precise trachomatous trichiasis prevalence estimates can be calculated from trachoma impact and surveillance prevalence surveys by borrowing the strength of information across space. The geostatistical approach therefore enables increased precision for less field-sampling effort.

Our geostatistical model (1) for the Ethiopia trachomatous trichiasis data included adjustments for year and for the varying demography of different evaluation units. It would be straightforward to extend the model to accommodate other forms of covariate information that can be made available throughout the area over which predictions are required, e.g. raster images of environmental variables.

The geostatistical approach is disease-agnostic. Its application to other neglected tropical diseases may involve including covariate information or incorporating established disease-specific knowledge to inform the specification of the residual spatial correlation structure. For an application to the design of elimination surveys for lymphatic filariasis in Ghana, see Fronterre *et al.*^50^

A perfect estimation of evaluation-unit-level prevalence would require adjusting for spatial heterogeneities in population size using high-quality, timely census data, available at every prediction geographical location. However, census data for Ethiopia are available as aggregated counts at the regional (admin two) level and not at fine spatial scales. Therefore, to account for spatial heterogeneities in population counts, we used population counts raster data from WorldPop. These data are modelled and are therefore a limitation of our analysis. A drawback of using these data is that in countries that have not had a census for a long time and where substantial subnational variations in migration, fertility and mortality exist, the WorldPop data can be highly uncertain. If estimates of possible uncertainties in these data were available, we could adjust for them by drawing sample population surfaces and recomputing evaluation-unit-level prevalence for each draw. By repeating this process a sufficiently large number of times, summaries of evaluation-unit-level prevalence would have considered possible uncertainties in the population estimates.

## Conclusions

The trachomatous trichiasis criterion for the elimination of trachoma as a public health problem, as defined by trachomatous trichiasis prevalence <0.002 in ≥15-year-olds, has not been widely achieved in Ethiopia. By exploiting residual spatial correlation in trachomatous trichiasis prevalence within an MBG framework, we obtained estimates of trachomatous trichiasis prevalence that are, on average, 10 times as precise as those obtained from the currently used approach. Geostatistical methods therefore present opportunities for sharper and more affordable sampling strategies to inform decisions on the elimination of trachoma and other neglected tropical diseases.

## Supplementary data

Supplementary data are available at *IJE* online.

## Ethics approval

Ethical clearance for the study was granted by the Lancaster University’s Faculty of Health and Medicine Research Ethics Committee (FHMREC21005).

## Funding

The authors acknowledge funding of the neglected tropical disease Modelling Consortium by the Bill and Melinda Gates Foundation [OPP1184344]. A.W.S. is a staff member of the World Health Organization.

## Disclaimer

The authors alone are responsible for the views expressed in this article and they do not necessarily represent the views, decisions or policies of the institutions with which they are affiliated. The boundaries and names shown and the designations used on the maps in this article do not imply the expression of any opinion whatsoever on the part of the authors, or the institutions with which they are affiliated, concerning the legal status of any country, territory, city or area or of its authorities, or concerning the delimitation of its frontiers or boundaries.

## Data availability

The data underlying this article were provided by the Federal Ministry of Health of Ethiopia (FMOH), with permission. Data will be shared on reasonable request to Fikre Seife, fikre-seife5@gmail.com, with the permission of the FMOH.

## Author contributions

A.W.S. and P.J.D. conceived of the project. All authors contributed to the formulation of overarching research goals and aims. B.A., E.G. and P.J.D. developed the geostatistical model and carried out the statistical analyses. B.A., C.F., O.J. and E.G. developed the code for the statistical analyses and carried out numerical simulations. N.N., F.S., M.D. and E.H.E. verified the traditional analysis. A.B. carried out data curation. B.A. produced visualization of the results and wrote the first draft. All authors provided critical feedback and helped to shape the research, analyses and manuscript.

## Conflict of interest

E.H.E. reports grants from the International Trachoma Initiative, Pfizer Inc., during the conduct of the study. A.B. reports personal fees from Pfizer Inc., outside the submitted work. For all the other authors, none declared.

## Supplementary Material

dyab227_Supplementary_Data
