# Correction to: Model-based geostatistics enables more precise estimates of neglected tropical-disease prevalence in elimination settings: mapping trachoma prevalence in Ethiopia

**DOI:** 10.1093/ije/dyac091

**Published:** 2022-05-10

**Authors:** Benjamin Amoah, Claudio Fronterre, Olatunji Johnson, Michael Dejene, Fikre Seife, Nebiyu Negussu, Ana Bakhtiari, Emma M Harding-Esch, Emanuele Giorgi, Anthony W Solomon, Peter J Diggle

*International Journal of Epidemiology*, 2021; dyab227, https://doi.org/10.1093/ije/dyab227

In the originally published version of this manuscript, a funding statement and disclaimer were omitted in error.

The following passages have been added to the Funding section:

1. a note reading “AWS is a staff member of the World Health Organization.”
2. a disclaimer reading, “The authors alone are responsible for the views expressed in this article and they do not necessarily represent the views, decisions or policies of the institutions with which they are affiliated. The boundaries and names shown and the designations used on the maps in this article do not imply the expression of any opinion whatsoever on the part of the authors, or the institutions with which they are affiliated, concerning the legal status of any country, territory, city or area or of its authorities, or concerning the delimitation of its frontiers or boundaries.”

These errors have been corrected online.

