# Supplementary material for: Model-based geostatistics enables more precise estimates of neglected tropical-disease prevalence in elimination settings: mapping trachoma prevalence in Ethiopia
Source: Int J Epidemiol. 2021 Nov 13;51(2):468–78. doi: 10.1093/ije/dyab227 (PMC9082807; doi:10.1093/ije/dyab227)
Supplement: dyab227_Supplementary_Data [file dyab227_supplementary_data.zip › ije-2020-07-1333-File008.pdf]

Supplementary Appendix S1: Details of the geostatistical modelling

This supplementary appendix gives details of the geostatistical modelling and spatial predictions.

Evidence of spatial correlation

Supplementary Figure 1 shows the empirical variogram of the estimated random effects from a generalised linear mixed model (GLMM) together with a variogram envelope generated under the assumption that there is no spatial correlation in the data. The empirical variogram lies mostly outside the envelope, indicating that the data are spatially correlated.

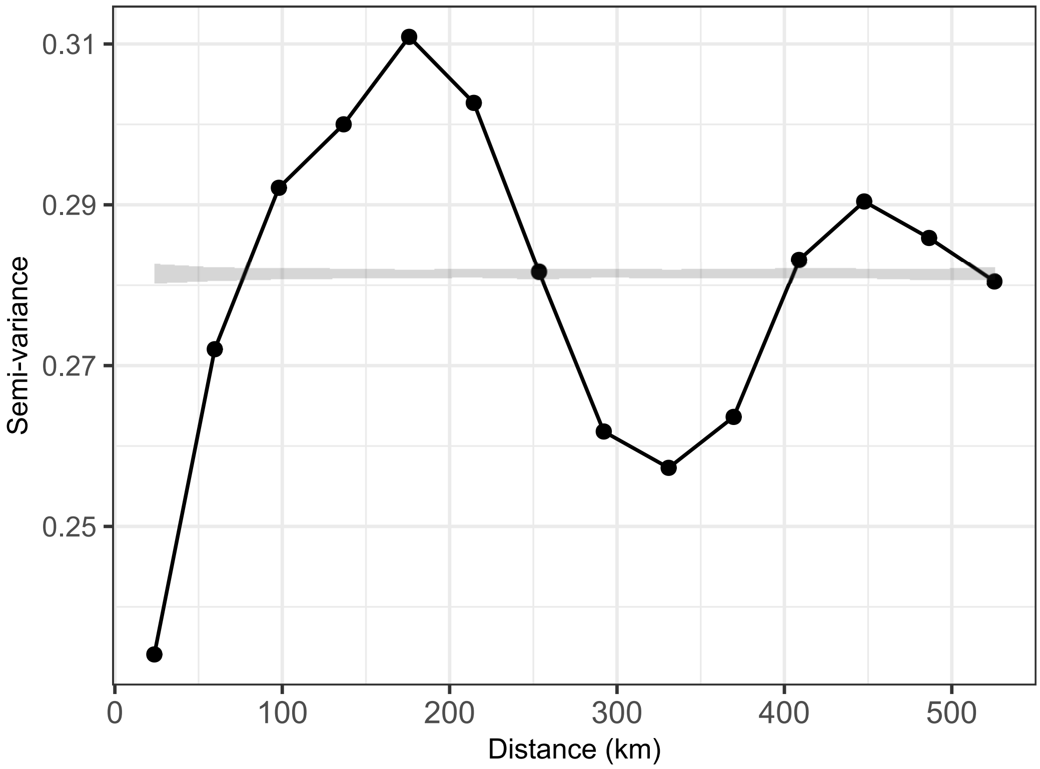

Appendix Figure 1. Empirical variogram of the residuals from a generalised linear mixed model (solid line) lying mostly outside of a variogram envelop (grey area) generated under the assumption of no spatial correlation in the data

Model specification

Let $p_{jkt}(x_i)$ represent the probability that an individual j belonging to an age-gender class k and living at cluster location x_i tests positive for TT in year t. Note that each i is implicitly a function of j, k and t.

We specify a geostatistical model as follows:

First, let $S(x)$ be a stationary and isotropic Gaussian process with mean zero, variance σ^2, and correlation

function $\rho(u) = \text{Corr}\{S(x), S(x-u)\} = \exp\{-u/\phi\}$, where the scale parameter ϕ regulates the rate at which the spatial correlation decays with increasing distance u. Second, Let Z_i be zero-mean independent and identically distributed Gaussian random variables with variance τ^2. Then conditional on $S(\cdot)$ and the Z_i, we model the probability of positive test as

$$\log\Big\{\frac{p_{jkt}(x_i)}{1-p_{jkt}(x_i)}\Big\} = \alpha_t + \beta_k + S(x_i) + Z_i. \quad \text{(Sup 1)}$$

We estimate the parameters of the model (Sup 1) by Monte Carlo maximum likelihood (MCML) as implemented in Prevmap, an R package,[1] freely available from the Comprehensive R Archive Network (`www.r-project.org`).

Spatial Prediction

For prediction, we denote by $\mathcal{A}$ our study-region, namely Ethiopia without Somali, and approximate this by a uniform grid of locations x^* at a spacing of 5km.

We first predict prevalence in 2019 for each age-gender at each prediction location, x^*, by using our fitted model to draw samples from the predictive distribution of $\mathcal{P}_k$, i.e. its conditional of prevalence in each age-gender class k given all available data. This can be formally expressed as

$$\mathcal{P}_k = \{P_k(x^*) : x^* \in \mathcal{A}, t = 2019\}. \quad \text{(Sup 2)}$$

We then compute age- and gender-standardized prevalence as

$$\mathcal{P} = \sum_k W_k(x^*) P_k(x^*), \quad \text{(Sup 3)}$$

where the weights $W_k(x^*)$ are local proportions of each age-gender class according to the Ethiopia census 2007 data, which are summarized in Supplementary Figure 2.

In the geostatistical approach we allow the true but unknown prevalence, $P(x)$,, to vary spatially. It follows that EU-wide prevalence is given by

$$\mathcal{P}(EU) = \int_{EU} pc(x)p(x)dx \Big/ \int_{EU} pc(x)dx, \quad \text{(Sup 4)}$$

where $pc(x)$ are the population counts (Supplementary Figure 3) obtained from WorldPop.[2]

Using the predictive samples for each EU, we obtain summary statistics such as mean prevalence, 95% confidence intervals and probability of elimination. The best (minimum mean square error) predictor of EU-wide prevalence is the sample mean of the sampled values of $\mathcal{P}$. The predictive probability of elimination is the proportion of sampled values less than 0.002.

An extended description of geostatistical methods and their application to global health research can be found elsewhere.[3,4]

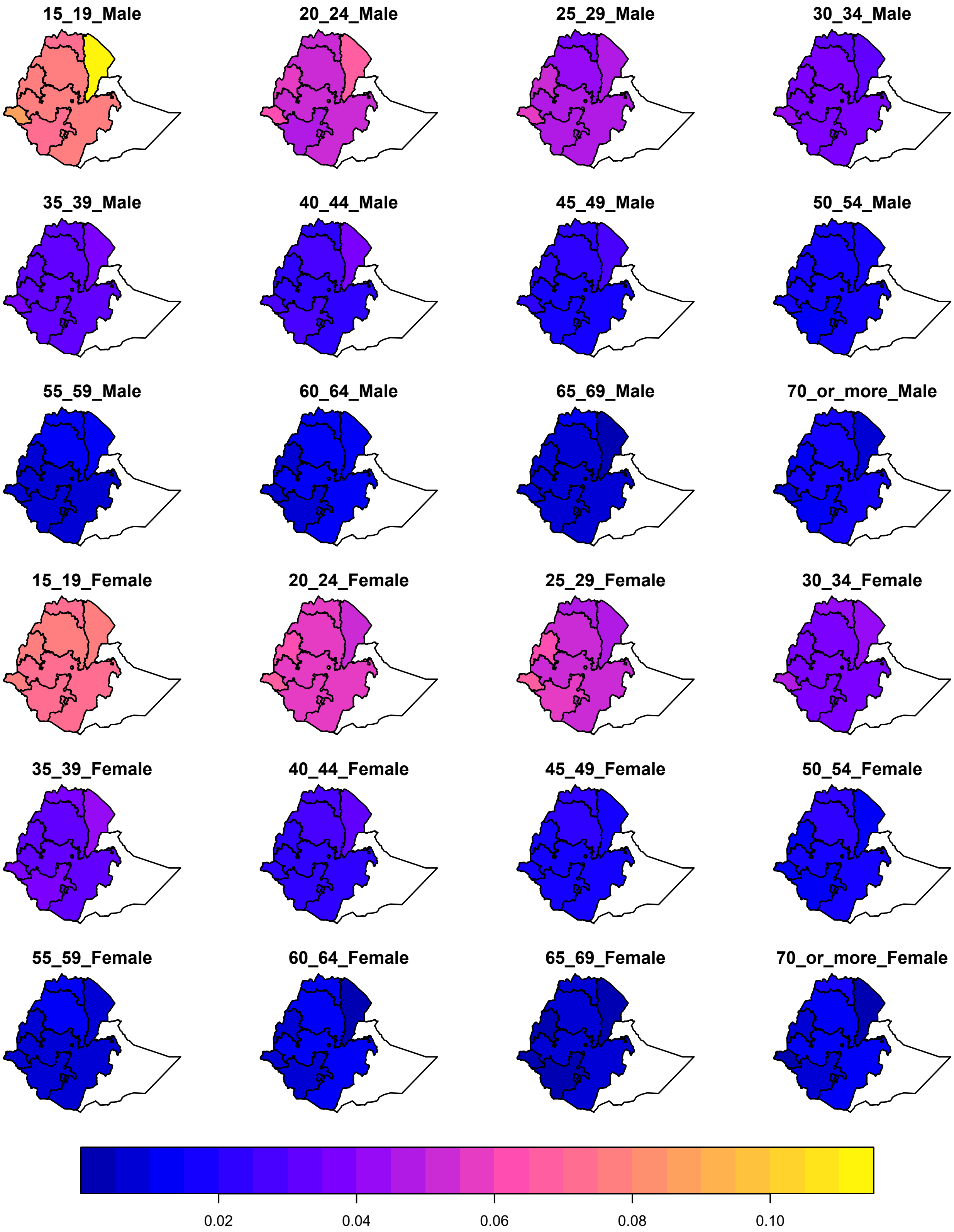

Appendix Figure 2. Map of the proportion of females and males in different age groups over the study region.

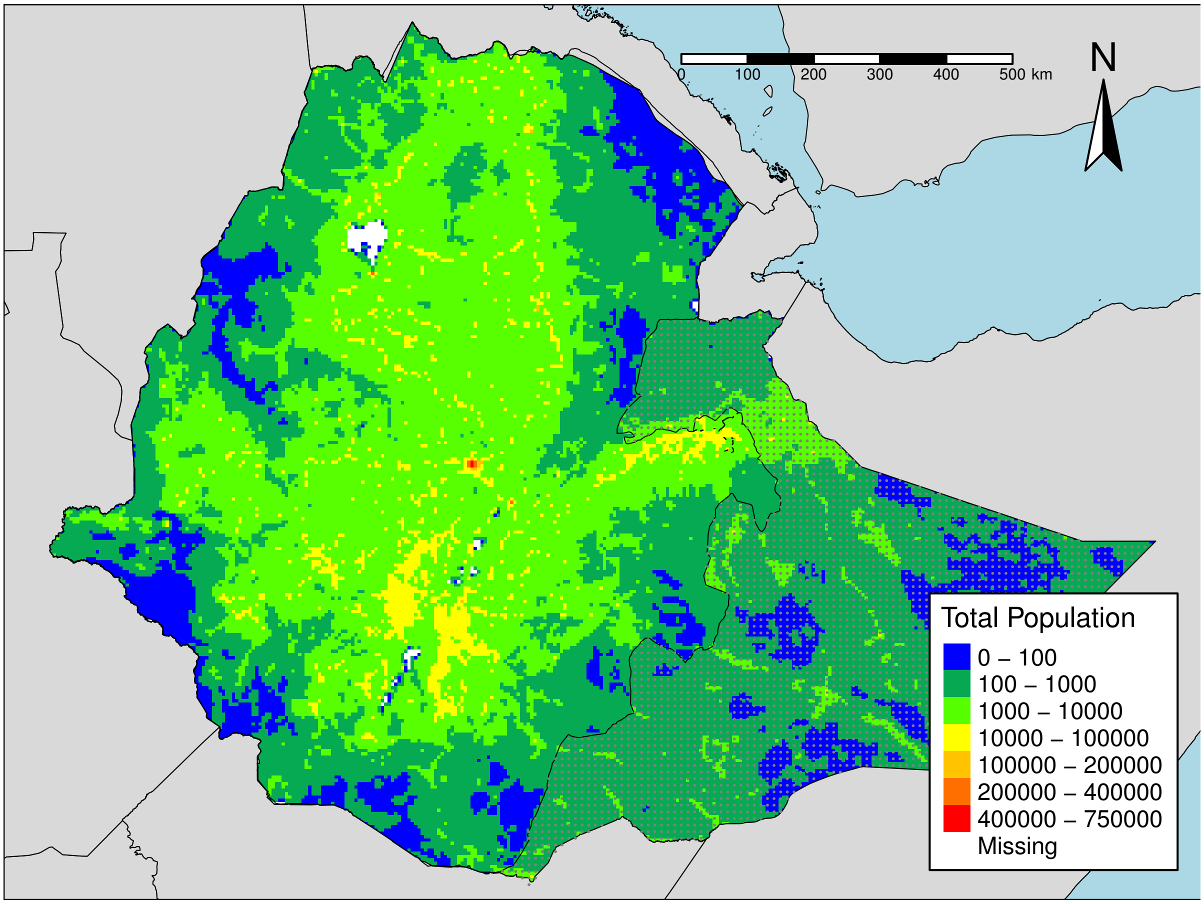

Appendix Figure 3. The population count data from WorldPop on a 5km grid

Model validation

We compared the empirical variogram of the data with a 95% tolerance envelope of empirical variograms from 1000 simulated datasets, each generated under the fitted model (Supplementary Figure 4).

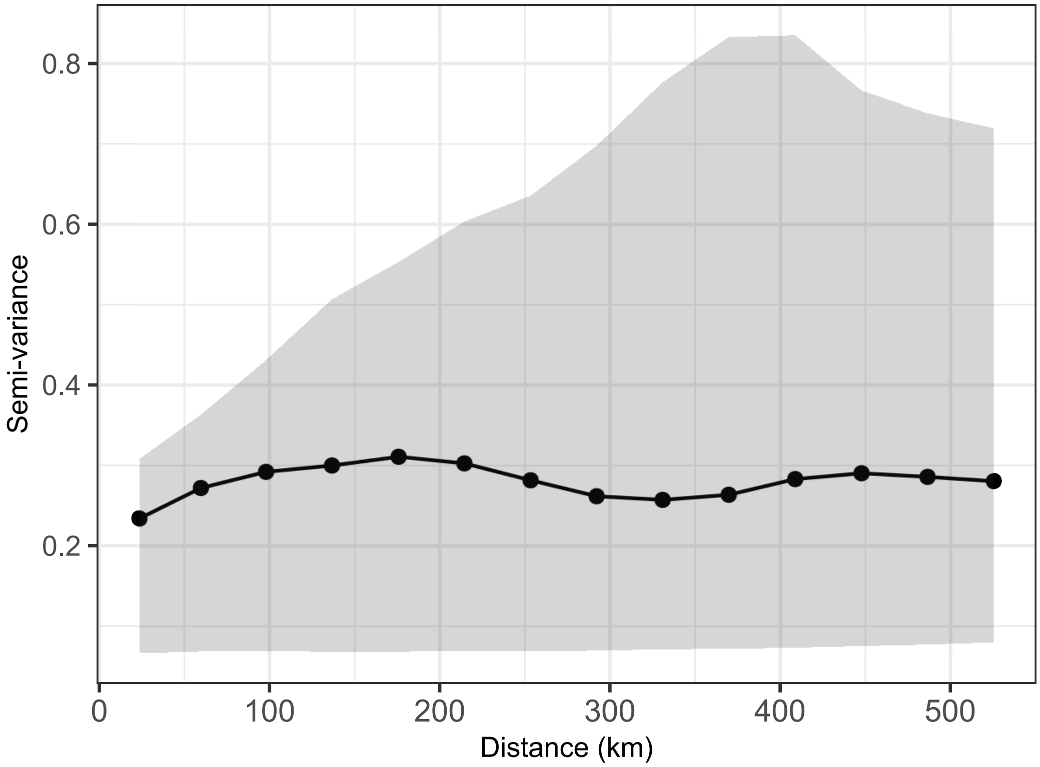

Appendix Figure 4. Results from the model validation. The solid line is the empirical variogram. The grey area is the 95% tolerance envelope of 1000 empirical variograms generated from simulations of the fitted model. The empirical variogram of the data lies entirely within the tolerance envelope showing that the fitted correlation structure is compatible with the data.

References

1 Giorgi E, Diggle P. Prevmap: an R package for prevalence mapping. Submitted; 2016.

2 Linard C, Gilbert M, Snow RW, Noor AM, Tatem AJ. Population distribution, settlement patterns and accessibility across Africa in 2010. PloS one. 2012;7(2):e31743.

3 Diggle PJ, Tawn JA, Moyeed R. Model-based geostatistics. Journal of the Royal Statistical Society: Series C (Applied Statistics). 1998;47(3):299–350.

4 Diggle PJ, Giorgi E. Model-based Geostatistics for Global Public Health: Methods and Applications. Chapman and Hall/CRC; 2019.
