# Supplementary material for: Model-based geostatistics enables more precise estimates of neglected tropical-disease prevalence in elimination settings: mapping trachoma prevalence in Ethiopia
Source: Int J Epidemiol. 2021 Nov 13;51(2):468–78. doi: 10.1093/ije/dyab227 (PMC9082807; doi:10.1093/ije/dyab227)
Supplement: dyab227_Supplementary_Data [file dyab227_supplementary_data.zip › ije-2020-07-1333-File009.pdf]

Supplementary Table S1: Details of the predictions from the geostatistical model showing EU names, estimated TT prevalence with associated 95% confidence intervals and the probability of elimination.

	Evalution Unit	Estimated TT prev.	95% Predictive Interval	Prob. of Elimination
1	Abala	0.0102	(0.0095, 0.0110)	0.0000
2	Abay Chomen	0.0114	(0.0107, 0.0121)	0.0000
3	Abe Dongoro	0.0080	(0.0074, 0.0086)	0.0000
4	Abichuna Gne'a	0.0050	(0.0047, 0.0053)	0.0000
5	Abobo	0.0153	(0.0144, 0.0161)	0.0000
6	Achefer	0.0117	(0.0111, 0.0123)	0.0000
7	Ada'a Chukala	0.0061	(0.0056, 0.0066)	0.0000
8	Adaba	0.0034	(0.0030, 0.0038)	0.0000
9	Adama	0.0087	(0.0078, 0.0095)	0.0000
10	Adami Tulu Jido Kombolcha	0.0064	(0.0058, 0.0070)	0.0000
11	Adda Berga	0.0035	(0.0033, 0.0038)	0.0000
12	Addi Arkay	0.0143	(0.0137, 0.0148)	0.0000
13	Adet	0.0172	(0.0164, 0.0180)	0.0000
14	Adolana Wadera	0.0023	(0.0020, 0.0026)	0.0386
15	Adwa	0.0146	(0.0138, 0.0155)	0.0000
16	Afambo	0.0065	(0.0060, 0.0069)	0.0000
17	Agarfa	0.0047	(0.0042, 0.0052)	0.0000
18	Agelo Meti	0.0045	(0.0042, 0.0049)	0.0000
19	Ahferom	0.0143	(0.0136, 0.0150)	0.0000
20	Akaki	0.0050	(0.0046, 0.0054)	0.0000
21	Akililna Mohr	0.0092	(0.0088, 0.0097)	0.0000
22	Akobo	0.0353	(0.0338, 0.0370)	0.0000
23	Alaba	0.0085	(0.0080, 0.0090)	0.0000
24	Alamata	0.0139	(0.0129, 0.0148)	0.0000
25	Ale	0.0019	(0.0018, 0.0021)	0.7592
26	Alefa	0.0105	(0.0100, 0.0110)	0.0000
27	Alem Gena	0.0040	(0.0037, 0.0043)	0.0000
28	Aleta Wendo	0.0020	(0.0019, 0.0021)	0.4823
29	Alicho Woriro	0.0063	(0.0059, 0.0067)	0.0000
30	Amaro	0.0047	(0.0043, 0.0050)	0.0000
31	Amba Sel	0.0096	(0.0091, 0.0100)	0.0000
32	Ambalaje	0.0171	(0.0160, 0.0183)	0.0000
33	Ambo	0.0034	(0.0032, 0.0036)	0.0000
34	Ameya	0.0071	(0.0067, 0.0074)	0.0000
35	Amibara	0.0093	(0.0088, 0.0099)	0.0000
36	Amigna	0.0069	(0.0064, 0.0075)	0.0000
37	Amuru	0.0117	(0.0110, 0.0125)	0.0000
38	Anchar	0.0106	(0.0099, 0.0114)	0.0000
39	Anderacha	0.0039	(0.0036, 0.0043)	0.0000
40	Anfilo	0.0097	(0.0089, 0.0106)	0.0000

	Evalution Unit	Estimated TT prev.	95% Predictive Interval	Prob. of Elimination
41	Angacha	0.0103	(0.0099, 0.0107)	0.0000
42	Angolela Tera	0.0056	(0.0054, 0.0059)	0.0000
43	Ankesha	0.0116	(0.0110, 0.0123)	0.0000
44	Ankober	0.0117	(0.0111, 0.0123)	0.0000
45	Antsokia Gemza	0.0100	(0.0096, 0.0104)	0.0000
46	Arba Minch Zuria	0.0055	(0.0052, 0.0058)	0.0000
47	Arbe Gona	0.0013	(0.0012, 0.0014)	1.0000
48	Arero	0.0051	(0.0046, 0.0055)	0.0000
49	Argoba Special	0.0096	(0.0091, 0.0102)	0.0000
50	Aroresa	0.0016	(0.0014, 0.0018)	1.0000
51	Arsi Negele	0.0053	(0.0048, 0.0057)	0.0000
52	Artuma	0.0066	(0.0061, 0.0071)	0.0000
53	Artuma Fursina	0.0106	(0.0102, 0.0110)	0.0000
54	Asagirt	0.0087	(0.0083, 0.0091)	0.0000
55	Asegede Tsimbela	0.0158	(0.0150, 0.0166)	0.0000
56	Aseko	0.0099	(0.0091, 0.0107)	0.0000
57	Asosa	0.0074	(0.0066, 0.0082)	0.0000
58	Atsbi Wenberta	0.0122	(0.0116, 0.0129)	0.0000
59	Awabel	0.0223	(0.0217, 0.0231)	0.0000
60	Awasa	0.0034	(0.0032, 0.0037)	0.0000
61	Awash Fentale	0.0094	(0.0088, 0.0101)	0.0000
62	Ayra Guliso	0.0041	(0.0038, 0.0045)	0.0000
63	Aysaita	0.0066	(0.0061, 0.0071)	0.0000
64	Badawacho	0.0126	(0.0121, 0.0130)	0.0000
65	Bahir Dar	0.0147	(0.0131, 0.0164)	0.0000
66	Bahir Dar Zuria	0.0145	(0.0137, 0.0153)	0.0000
67	Bako Gazer	0.0052	(0.0047, 0.0058)	0.0000
68	Bako Tibe	0.0053	(0.0049, 0.0057)	0.0000
69	Bambasi	0.0069	(0.0063, 0.0075)	0.0000
70	Banja	0.0118	(0.0112, 0.0125)	0.0000
71	Basketo	0.0053	(0.0048, 0.0058)	0.0000
72	Baso Liben	0.0286	(0.0276, 0.0296)	0.0000
73	Bati	0.0087	(0.0083, 0.0091)	0.0000
74	Becho	0.0064	(0.0060, 0.0068)	0.0000
75	Bedele	0.0065	(0.0062, 0.0069)	0.0000
76	Bedeno	0.0081	(0.0074, 0.0088)	0.0000
77	Begi	0.0082	(0.0076, 0.0089)	0.0000
78	Bekoji	0.0044	(0.0040, 0.0048)	0.0000
79	Belesa	0.0168	(0.0160, 0.0177)	0.0000
80	Belo Jegonfoy	0.0049	(0.0046, 0.0053)	0.0000

	Evalution Unit	Estimated TT prev.	95% Predictive Interval	Prob. of Elimination
81	Bena	0.0059	(0.0054, 0.0066)	0.0000
82	Bench	0.0059	(0.0053, 0.0064)	0.0000
83	Bensa	0.0014	(0.0013, 0.0016)	1.0000
84	Berahle	0.0073	(0.0068, 0.0078)	0.0000
85	Berbere	0.0041	(0.0037, 0.0046)	0.0000
86	Berehet	0.0115	(0.0108, 0.0121)	0.0000
87	Berehna Aleltu	0.0033	(0.0031, 0.0036)	0.0000
88	Bero	0.0086	(0.0073, 0.0099)	0.0000
89	Beyeda	0.0117	(0.0111, 0.0122)	0.0000
90	Bibugn	0.0144	(0.0139, 0.0150)	0.0000
91	Bila Seyo	0.0060	(0.0056, 0.0065)	0.0000
92	Bita	0.0044	(0.0040, 0.0048)	0.0000
93	Boji	0.0043	(0.0040, 0.0047)	0.0000
94	Boke	0.0093	(0.0087, 0.0099)	0.0000
95	Boloso Sore	0.0189	(0.0183, 0.0194)	0.0000
96	Bonke	0.0056	(0.0053, 0.0059)	0.0000
97	Bore	0.0017	(0.0015, 0.0019)	0.9998
98	Borecha	0.0070	(0.0065, 0.0074)	0.0000
99	Boreda	0.0060	(0.0058, 0.0063)	0.0000
100	Boset	0.0106	(0.0098, 0.0114)	0.0000
101	Bugna	0.0190	(0.0184, 0.0196)	0.0000
102	Bule	0.0020	(0.0018, 0.0022)	0.5007
103	Bulen	0.0051	(0.0047, 0.0056)	0.0000
104	Bure	0.0027	(0.0025, 0.0030)	0.0000
105	Bure Mudaytu	0.0073	(0.0069, 0.0077)	0.0000
106	Bure Wemberma	0.0132	(0.0124, 0.0139)	0.0000
107	Burji	0.0051	(0.0047, 0.0056)	0.0000
108	Cheha	0.0213	(0.0207, 0.0219)	0.0000
109	Cheliya	0.0045	(0.0042, 0.0048)	0.0000
110	Chena	0.0048	(0.0044, 0.0052)	0.0000
111	Chencha	0.0053	(0.0051, 0.0055)	0.0000
112	Cheta	0.0056	(0.0051, 0.0061)	0.0000
113	Chifra	0.0065	(0.0061, 0.0069)	0.0000
114	Chilga	0.0115	(0.0108, 0.0122)	0.0000
115	Chiro	0.0100	(0.0093, 0.0107)	0.0000
116	Chole	0.0083	(0.0076, 0.0090)	0.0000
117	Chora	0.0048	(0.0045, 0.0051)	0.0000
118	Dabat	0.0135	(0.0128, 0.0143)	0.0000
119	Dale	0.0026	(0.0024, 0.0028)	0.0000
120	Dale Sadi	0.0039	(0.0035, 0.0043)	0.0000

	Evalution Unit	Estimated TT prev.	95% Predictive Interval	Prob. of Elimination
121	Dallol	0.0058	(0.0055, 0.0062)	0.0000
122	Dalocha	0.0073	(0.0069, 0.0077)	0.0000
123	Damot Gale	0.0175	(0.0170, 0.0181)	0.0000
124	Damot Weyde	0.0109	(0.0105, 0.0113)	0.0000
125	Dangila	0.0106	(0.0102, 0.0111)	0.0000
126	Dangur	0.0076	(0.0071, 0.0082)	0.0000
127	Dano	0.0043	(0.0040, 0.0046)	0.0000
128	Dara	0.0018	(0.0016, 0.0020)	0.9916
129	Daramalo	0.0059	(0.0056, 0.0063)	0.0000
130	Darimu	0.0025	(0.0023, 0.0028)	0.0000
131	Darolebu	0.0089	(0.0084, 0.0095)	0.0000
132	Dawa Chefa	0.0116	(0.0112, 0.0121)	0.0000
133	Dawo	0.0051	(0.0047, 0.0055)	0.0000
134	Dawunt Delanta	0.0112	(0.0107, 0.0116)	0.0000
135	Debarq	0.0131	(0.0126, 0.0137)	0.0000
136	Debay Telatgen	0.0133	(0.0128, 0.0139)	0.0000
137	Debre Berhan Zuria	0.0078	(0.0074, 0.0081)	0.0000
138	Debre Marqos	0.0180	(0.0170, 0.0191)	0.0000
139	Debre Sina	0.0145	(0.0138, 0.0152)	0.0000
140	Debre Tabor	0.0103	(0.0095, 0.0111)	0.0000
141	Decha	0.0053	(0.0049, 0.0057)	0.0000
142	Deder	0.0084	(0.0077, 0.0091)	0.0000
143	Dedesa	0.0066	(0.0060, 0.0071)	0.0000
144	Dedo	0.0074	(0.0069, 0.0080)	0.0000
145	Dega	0.0050	(0.0046, 0.0054)	0.0000
146	Dega Damot	0.0128	(0.0123, 0.0135)	0.0000
147	Degeluna Tijo	0.0055	(0.0050, 0.0061)	0.0000
148	Degem	0.0084	(0.0079, 0.0088)	0.0000
149	Degua Temben	0.0182	(0.0171, 0.0193)	0.0000
150	Dehana	0.0215	(0.0206, 0.0225)	0.0000
151	Dejen	0.0277	(0.0268, 0.0287)	0.0000
152	Dembecha	0.0159	(0.0150, 0.0168)	0.0000
153	Dembia	0.0141	(0.0134, 0.0148)	0.0000
154	Dendi	0.0033	(0.0032, 0.0035)	0.0000
155	Dera	0.0175	(0.0169, 0.0180)	0.0000
156	Dessie	0.0100	(0.0093, 0.0107)	0.0000
157	Dessie Zuria	0.0098	(0.0094, 0.0102)	0.0000
158	Dewe	0.0069	(0.0065, 0.0073)	0.0000
159	Dibate	0.0064	(0.0059, 0.0070)	0.0000
160	Diga	0.0069	(0.0064, 0.0073)	0.0000

	Evalution Unit	Estimated TT prev.	95% Predictive Interval	Prob. of Elimination
161	Dima	0.0066	(0.0059, 0.0075)	0.0000
162	Dirashe	0.0061	(0.0057, 0.0065)	0.0000
163	Dire	0.0067	(0.0062, 0.0072)	0.0000
164	Dire Dawa	0.0067	(0.0061, 0.0073)	0.0000
165	Dita	0.0054	(0.0052, 0.0057)	0.0000
166	Doba	0.0094	(0.0087, 0.0101)	0.0000
167	Dodola	0.0025	(0.0022, 0.0028)	0.0000
168	Dodotana Sire	0.0083	(0.0077, 0.0090)	0.0000
169	Dubti	0.0068	(0.0064, 0.0072)	0.0000
170	Dugda Bora	0.0062	(0.0057, 0.0068)	0.0000
171	Dulecha	0.0100	(0.0095, 0.0106)	0.0000
172	Ebenat	0.0160	(0.0152, 0.0168)	0.0000
173	Efratana Gidim	0.0111	(0.0107, 0.0116)	0.0000
174	Ejere (Addis Alem)	0.0029	(0.0027, 0.0031)	0.0000
175	Ela (Konta)	0.0061	(0.0057, 0.0065)	0.0000
176	Enarj Enawga	0.0215	(0.0207, 0.0222)	0.0000
177	Enbise Sar Midir	0.0268	(0.0258, 0.0278)	0.0000
178	Endagagn	0.0086	(0.0081, 0.0091)	0.0000
179	Endamehoni	0.0164	(0.0152, 0.0177)	0.0000
180	Enderta	0.0210	(0.0192, 0.0231)	0.0000
181	Enemay	0.0234	(0.0226, 0.0243)	0.0000
182	Enemorina Eaner	0.0182	(0.0176, 0.0188)	0.0000
183	Erebti	0.0082	(0.0077, 0.0088)	0.0000
184	Erob	0.0088	(0.0083, 0.0094)	0.0000
185	Esite	0.0191	(0.0184, 0.0198)	0.0000
186	Ewa	0.0073	(0.0069, 0.0078)	0.0000
187	Ezha	0.0131	(0.0127, 0.0136)	0.0000
188	Fagta Lakoma	0.0112	(0.0107, 0.0118)	0.0000
189	Farta	0.0115	(0.0109, 0.0121)	0.0000
190	Fedis	0.0070	(0.0064, 0.0076)	0.0000
191	Fentale	0.0110	(0.0102, 0.0119)	0.0000
192	Fogera	0.0132	(0.0126, 0.0139)	0.0000
193	Fursi	0.0071	(0.0067, 0.0075)	0.0000
194	Gambela	0.0103	(0.0094, 0.0113)	0.0000
195	Ganji	0.0036	(0.0033, 0.0040)	0.0000
196	Ganta Afeshum	0.0136	(0.0129, 0.0142)	0.0000
197	Gasera	0.0054	(0.0049, 0.0060)	0.0000
198	Gawo Dale	0.0051	(0.0047, 0.0055)	0.0000
199	Gechi	0.0065	(0.0061, 0.0069)	0.0000
200	Gedeb	0.0033	(0.0030, 0.0037)	0.0000

	Evalution Unit	Estimated TT prev.	95% Predictive Interval	Prob. of Elimination
201	Gelana Abaya	0.0032	(0.0029, 0.0035)	0.0000
202	Gelila	0.0047	(0.0043, 0.0051)	0.0000
203	Gena Bosa	0.0103	(0.0097, 0.0110)	0.0000
204	Gera	0.0048	(0.0044, 0.0052)	0.0000
205	Gera Midirna Keya Gabriel	0.0112	(0.0108, 0.0115)	0.0000
206	Gerar Jarso	0.0087	(0.0082, 0.0091)	0.0000
207	Gesha Daka	0.0031	(0.0028, 0.0034)	0.0000
208	Gewane	0.0071	(0.0067, 0.0075)	0.0000
209	Gewata	0.0037	(0.0034, 0.0040)	0.0000
210	Gida Kiremu	0.0086	(0.0080, 0.0092)	0.0000
211	Gidami	0.0093	(0.0086, 0.0100)	0.0000
212	Gidan	0.0093	(0.0089, 0.0097)	0.0000
213	Gimbi	0.0043	(0.0039, 0.0047)	0.0000
214	Gimbichu	0.0064	(0.0060, 0.0068)	0.0000
215	Gimbo	0.0049	(0.0045, 0.0053)	0.0000
216	Ginde Beret	0.0086	(0.0083, 0.0090)	0.0000
217	Ginir	0.0058	(0.0053, 0.0063)	0.0000
218	Girawa	0.0077	(0.0071, 0.0084)	0.0000
219	Gishe Rabel	0.0088	(0.0084, 0.0092)	0.0000
220	Goba	0.0040	(0.0035, 0.0045)	0.0000
221	Goba Koricha	0.0108	(0.0100, 0.0116)	0.0000
222	Godere	0.0050	(0.0046, 0.0055)	0.0000
223	Gofa Zuria	0.0054	(0.0050, 0.0058)	0.0000
224	Gog	0.0236	(0.0221, 0.0253)	0.0000
225	Golo Odo	0.0084	(0.0077, 0.0090)	0.0000
226	Gololcha	0.0081	(0.0075, 0.0086)	0.0000
227	Goma	0.0066	(0.0060, 0.0071)	0.0000
228	Goncha Siso Enese	0.0218	(0.0210, 0.0227)	0.0000
229	Gonder	0.0121	(0.0115, 0.0128)	0.0000
230	Gonder Zuria	0.0139	(0.0133, 0.0145)	0.0000
231	Goro	0.0108	(0.0104, 0.0111)	0.0000
232	Goro Gutu	0.0084	(0.0077, 0.0091)	0.0000
233	Guangua	0.0101	(0.0095, 0.0106)	0.0000
234	Guba	0.0074	(0.0065, 0.0083)	0.0000
235	Guba Lafto	0.0097	(0.0093, 0.0101)	0.0000
236	Guduru	0.0092	(0.0087, 0.0097)	0.0000
237	Gulina	0.0078	(0.0073, 0.0083)	0.0000
238	Gulomahda	0.0127	(0.0121, 0.0134)	0.0000
239	Gumer	0.0085	(0.0081, 0.0088)	0.0000
240	Guradamole	0.0047	(0.0043, 0.0052)	0.0000

	Evalution Unit	Estimated TT prev.	95% Predictive Interval	Prob. of Elimination
241	Guto Wayu	0.0079	(0.0075, 0.0084)	0.0000
242	Guzamn	0.0168	(0.0162, 0.0173)	0.0000
243	Habro	0.0104	(0.0097, 0.0112)	0.0000
244	Habru	0.0073	(0.0069, 0.0078)	0.0000
245	Hagere Mariam	0.0035	(0.0032, 0.0038)	0.0000
246	Hagere Mariamna Kesem	0.0075	(0.0071, 0.0079)	0.0000
247	Hamer	0.0070	(0.0063, 0.0077)	0.0000
248	Harar/Hundene	0.0063	(0.0057, 0.0069)	0.0000
249	Haro Maya	0.0058	(0.0053, 0.0062)	0.0000
250	Haru	0.0039	(0.0035, 0.0043)	0.0000
251	Hawa Welele	0.0053	(0.0049, 0.0059)	0.0000
252	Hawzen	0.0186	(0.0178, 0.0194)	0.0000
253	Hidabu Abote	0.0117	(0.0111, 0.0124)	0.0000
254	Hintalo Wajirat	0.0181	(0.0169, 0.0193)	0.0000
255	Hitosa	0.0071	(0.0065, 0.0077)	0.0000
256	Hulet Ej Enese	0.0169	(0.0163, 0.0175)	0.0000
257	Hulla	0.0013	(0.0012, 0.0015)	1.0000
258	Humbo	0.0085	(0.0080, 0.0090)	0.0000
259	Ibantu	0.0075	(0.0069, 0.0082)	0.0000
260	Ilu	0.0050	(0.0046, 0.0054)	0.0000
261	Isara	0.0067	(0.0063, 0.0072)	0.0000
262	Itang	0.0208	(0.0198, 0.0219)	0.0000
263	Jabi Tehnan	0.0159	(0.0151, 0.0167)	0.0000
264	Jama	0.0169	(0.0162, 0.0175)	0.0000
265	Janamora	0.0147	(0.0140, 0.0153)	0.0000
266	Jarso	0.0054	(0.0050, 0.0058)	0.0000
267	Jarti	0.0116	(0.0109, 0.0123)	0.0000
268	Jeju	0.0090	(0.0083, 0.0097)	0.0000
269	Jeldu	0.0038	(0.0036, 0.0040)	0.0000
270	Jikawo	0.0256	(0.0244, 0.0268)	0.0000
271	Jile Timuga	0.0114	(0.0108, 0.0121)	0.0000
272	Jimma Arjo	0.0084	(0.0080, 0.0089)	0.0000
273	Jimma Horo	0.0082	(0.0076, 0.0088)	0.0000
274	Jimma Rare	0.0057	(0.0052, 0.0061)	0.0000
275	JimmaHoro	0.0070	(0.0064, 0.0077)	0.0000
276	Jor	0.0341	(0.0322, 0.0362)	0.0000
277	Kacha Bira	0.0136	(0.0131, 0.0141)	0.0000
278	Kafta Humera	0.0114	(0.0104, 0.0125)	0.0000
279	Kalu	0.0105	(0.0102, 0.0109)	0.0000
280	Kamashi	0.0043	(0.0040, 0.0046)	0.0000

	Evalution Unit	Estimated TT prev.	95% Predictive Interval	Prob. of Elimination
281	Kedida Gamela	0.0096	(0.0092, 0.0100)	0.0000
282	Kelela	0.0160	(0.0154, 0.0167)	0.0000
283	Kemba	0.0054	(0.0051, 0.0058)	0.0000
284	Kembibit	0.0032	(0.0030, 0.0035)	0.0000
285	Kemekem	0.0146	(0.0139, 0.0153)	0.0000
286	Kersa	0.0071	(0.0067, 0.0075)	0.0000
287	Kersana Kondaltiti	0.0062	(0.0057, 0.0066)	0.0000
288	Kewet	0.0118	(0.0113, 0.0124)	0.0000
289	Kindo Koysha	0.0110	(0.0106, 0.0115)	0.0000
290	Kobo	0.0109	(0.0104, 0.0114)	0.0000
291	Kochere	0.0029	(0.0027, 0.0032)	0.0000
292	Kofele	0.0030	(0.0027, 0.0033)	0.0000
293	Kokir	0.0071	(0.0067, 0.0076)	0.0000
294	Kokir Gedbano Gutazer	0.0079	(0.0075, 0.0084)	0.0000
295	Kokosa	0.0018	(0.0016, 0.0019)	0.9954
296	Kola Temben	0.0174	(0.0165, 0.0183)	0.0000
297	Kombolcha	0.0053	(0.0048, 0.0059)	0.0000
298	Komesha	0.0075	(0.0068, 0.0083)	0.0000
299	Koneba	0.0067	(0.0062, 0.0072)	0.0000
300	Konso	0.0062	(0.0057, 0.0068)	0.0000
301	Kucha	0.0063	(0.0060, 0.0066)	0.0000
302	Kuni	0.0098	(0.0091, 0.0105)	0.0000
303	Kuraz	0.0081	(0.0074, 0.0089)	0.0000
304	Kurfa Chele	0.0072	(0.0066, 0.0078)	0.0000
305	Kurmuk	0.0095	(0.0086, 0.0105)	0.0000
306	Kutaber	0.0088	(0.0083, 0.0092)	0.0000
307	Kuyu	0.0076	(0.0072, 0.0080)	0.0000
308	Laelay Adiyabo	0.0142	(0.0134, 0.0150)	0.0000
309	Laelay Maychew	0.0153	(0.0143, 0.0163)	0.0000
310	Lalo Asabi	0.0041	(0.0037, 0.0045)	0.0000
311	LaloKillie	0.0033	(0.0030, 0.0037)	0.0000
312	Lanfero	0.0069	(0.0063, 0.0074)	0.0000
313	Lay Armacheho	0.0099	(0.0094, 0.0105)	0.0000
314	Lay Betna Tach Bet	0.0237	(0.0229, 0.0245)	0.0000
315	Lay Gayint	0.0152	(0.0144, 0.0161)	0.0000
316	Legambo	0.0084	(0.0080, 0.0089)	0.0000
317	Leka Dulcha	0.0101	(0.0096, 0.0106)	0.0000
318	Liben	0.0037	(0.0032, 0.0041)	0.0000
319	Limu	0.0083	(0.0080, 0.0087)	0.0000
320	Limu Kosa	0.0059	(0.0056, 0.0063)	0.0000

	Evalution Unit	Estimated TT prev.	95% Predictive Interval	Prob. of Elimination
321	Limu Seka	0.0047	(0.0045, 0.0051)	0.0000
322	Loma Bosa	0.0093	(0.0088, 0.0097)	0.0000
323	Lome	0.0080	(0.0074, 0.0087)	0.0000
324	Machakel	0.0164	(0.0158, 0.0171)	0.0000
325	Maji	0.0074	(0.0066, 0.0083)	0.0000
326	Malka Balo	0.0088	(0.0081, 0.0095)	0.0000
327	Mama Midirna Lalo	0.0089	(0.0086, 0.0093)	0.0000
328	Mana	0.0071	(0.0065, 0.0078)	0.0000
329	Mana Sibu	0.0061	(0.0056, 0.0067)	0.0000
330	Mandura	0.0078	(0.0073, 0.0084)	0.0000
331	Mareka Gena	0.0085	(0.0079, 0.0091)	0.0000
332	Masha	0.0027	(0.0025, 0.0030)	0.0000
333	Meanit Goldiiya	0.0058	(0.0053, 0.0064)	0.0000
334	Meda Welabu	0.0033	(0.0029, 0.0037)	0.0000
335	Medebay Zana	0.0166	(0.0159, 0.0173)	0.0000
336	Megale	0.0096	(0.0091, 0.0103)	0.0000
337	Mekdela	0.0123	(0.0116, 0.0129)	0.0000
338	Mekele	0.0220	(0.0197, 0.0246)	0.0000
339	Meket	0.0142	(0.0137, 0.0149)	0.0000
340	Melekoza	0.0056	(0.0052, 0.0061)	0.0000
341	Menge	0.0075	(0.0069, 0.0083)	0.0000
342	Menit Shasha	0.0068	(0.0061, 0.0075)	0.0000
343	Menjiwo	0.0057	(0.0053, 0.0061)	0.0000
344	Mennana Arena Buluk	0.0030	(0.0027, 0.0034)	0.0000
345	Merawi	0.0131	(0.0124, 0.0137)	0.0000
346	Mereb Lehe	0.0121	(0.0114, 0.0129)	0.0000
347	Merti	0.0098	(0.0091, 0.0106)	0.0000
348	Mesela	0.0092	(0.0085, 0.0099)	0.0000
349	Meskanena Mareko	0.0052	(0.0048, 0.0056)	0.0000
350	Meta	0.0076	(0.0070, 0.0083)	0.0000
351	Meta Robi	0.0042	(0.0039, 0.0044)	0.0000
352	Metema	0.0126	(0.0117, 0.0135)	0.0000
353	Metu	0.0019	(0.0017, 0.0020)	0.9444
354	Meyu	0.0079	(0.0073, 0.0085)	0.0000
355	Mieso	0.0108	(0.0102, 0.0115)	0.0000
356	Mille	0.0063	(0.0060, 0.0067)	0.0000
357	Minjarna Shenkora	0.0129	(0.0122, 0.0135)	0.0000
358	MirabAbaya	0.0055	(0.0052, 0.0058)	0.0000
359	Misha	0.0106	(0.0102, 0.0111)	0.0000
360	Mojana Wadera	0.0104	(0.0099, 0.0110)	0.0000

	Evalution Unit	Estimated TT prev.	95% Predictive Interval	Prob. of Elimination
361	Moretna Jiru	0.0196	(0.0189, 0.0203)	0.0000
362	Moyale	0.0068	(0.0062, 0.0075)	0.0000
363	Mulona Sululta	0.0026	(0.0024, 0.0028)	0.0000
364	Munessa	0.0050	(0.0045, 0.0055)	0.0000
365	Naeder Adet	0.0190	(0.0180, 0.0200)	0.0000
366	Nejo	0.0049	(0.0045, 0.0054)	0.0000
367	Nenesebo	0.0020	(0.0017, 0.0022)	0.6291
368	Nole Kaba	0.0032	(0.0029, 0.0035)	0.0000
369	Nono	0.0049	(0.0047, 0.0052)	0.0000
370	Nunu Kumba	0.0066	(0.0062, 0.0070)	0.0000
371	Oda Godere	0.0058	(0.0053, 0.0064)	0.0000
372	Odo Shakiso	0.0029	(0.0026, 0.0032)	0.0000
373	Ofa	0.0083	(0.0079, 0.0087)	0.0000
374	Ofla	0.0155	(0.0145, 0.0165)	0.0000
375	Omo Sheleko	0.0169	(0.0163, 0.0175)	0.0000
376	Omonada	0.0086	(0.0081, 0.0091)	0.0000
377	Pawe	0.0077	(0.0071, 0.0082)	0.0000
378	Quara	0.0111	(0.0103, 0.0120)	0.0000
379	Quarit	0.0156	(0.0149, 0.0163)	0.0000
380	Raya Azebo	0.0152	(0.0142, 0.0162)	0.0000
381	Robe	0.0062	(0.0056, 0.0067)	0.0000
382	Saesi Tsaedaemba	0.0132	(0.0126, 0.0138)	0.0000
383	Samre	0.0170	(0.0160, 0.0182)	0.0000
384	Sanja	0.0106	(0.0101, 0.0111)	0.0000
385	Sasiga	0.0065	(0.0060, 0.0070)	0.0000
386	Sayint	0.0113	(0.0108, 0.0119)	0.0000
387	Sayo	0.0065	(0.0059, 0.0071)	0.0000
388	Seka Chekorsa	0.0065	(0.0060, 0.0070)	0.0000
389	Sekela	0.0130	(0.0124, 0.0137)	0.0000
390	Sekoru	0.0121	(0.0116, 0.0127)	0.0000
391	Sekota	0.0164	(0.0158, 0.0170)	0.0000
392	Selamgo	0.0060	(0.0055, 0.0066)	0.0000
393	Selti	0.0060	(0.0056, 0.0064)	0.0000
394	Seru	0.0065	(0.0060, 0.0071)	0.0000
395	Setema	0.0048	(0.0044, 0.0052)	0.0000
396	Shashemene	0.0043	(0.0039, 0.0047)	0.0000
397	Shebedino	0.0035	(0.0033, 0.0038)	0.0000
398	Shebel Berenta	0.0244	(0.0234, 0.0253)	0.0000
399	Sheka	0.0070	(0.0063, 0.0077)	0.0000
400	Sherkole	0.0093	(0.0084, 0.0101)	0.0000

	Evalution Unit	Estimated TT prev.	95% Predictive Interval	Prob. of Elimination
401	Shewa Bench	0.0058	(0.0053, 0.0064)	0.0000
402	Shirka	0.0052	(0.0047, 0.0057)	0.0000
403	Sibu Sire	0.0063	(0.0059, 0.0066)	0.0000
404	Sigmo	0.0035	(0.0032, 0.0039)	0.0000
405	Simada	0.0190	(0.0181, 0.0198)	0.0000
406	Simurobi Gele'alo	0.0086	(0.0081, 0.0091)	0.0000
407	Sinanana Dinsho	0.0045	(0.0041, 0.0050)	0.0000
408	Siraro	0.0075	(0.0071, 0.0080)	0.0000
409	Sirba Abay	0.0053	(0.0049, 0.0058)	0.0000
410	Siya Debirna Wayu & Ensaro	0.0126	(0.0121, 0.0131)	0.0000
411	Sodo	0.0055	(0.0050, 0.0059)	0.0000
412	Sodo Zuria	0.0158	(0.0151, 0.0164)	0.0000
413	Soro	0.0128	(0.0124, 0.0133)	0.0000
414	Sude	0.0074	(0.0069, 0.0080)	0.0000
415	Supena Sodo	0.0027	(0.0025, 0.0030)	0.0000
416	Surma	0.0089	(0.0078, 0.0101)	0.0000
417	Sylem	0.0025	(0.0022, 0.0028)	0.0000
418	Tach Gayint	0.0171	(0.0163, 0.0180)	0.0000
419	Tahtay Adiyabo	0.0118	(0.0110, 0.0127)	0.0000
420	Tahtay Koraro	0.0174	(0.0164, 0.0183)	0.0000
421	Tahtay Maychew	0.0155	(0.0146, 0.0164)	0.0000
422	Tanku Abergele	0.0144	(0.0136, 0.0152)	0.0000
423	Tarmaber	0.0105	(0.0101, 0.0110)	0.0000
424	Tehuledere	0.0100	(0.0096, 0.0104)	0.0000
425	Telalak	0.0064	(0.0060, 0.0068)	0.0000
426	Telo	0.0055	(0.0051, 0.0059)	0.0000
427	Teltele	0.0069	(0.0063, 0.0075)	0.0000
428	Tena	0.0064	(0.0059, 0.0070)	0.0000
429	Tenta	0.0100	(0.0095, 0.0105)	0.0000
430	Teru	0.0081	(0.0076, 0.0087)	0.0000
431	Tikur	0.0034	(0.0032, 0.0037)	0.0000
432	Tiro Afeta	0.0085	(0.0080, 0.0090)	0.0000
433	Tiyo	0.0061	(0.0054, 0.0068)	0.0000
434	Tocha	0.0073	(0.0069, 0.0078)	0.0000
435	Tole	0.0063	(0.0059, 0.0068)	0.0000
436	Tongo Sp. Wereda	0.0092	(0.0086, 0.0100)	0.0000
437	Tsegede	0.0095	(0.0089, 0.0101)	0.0000
438	Tsilemti	0.0177	(0.0169, 0.0185)	0.0000
439	Tulo	0.0093	(0.0086, 0.0101)	0.0000
440	Ubadebretsehay	0.0048	(0.0044, 0.0051)	0.0000

	Evalution Unit	Estimated TT prev.	95% Predictive Interval	Prob. of Elimination
441	Uraga	0.0022	(0.0020, 0.0024)	0.0572
442	Wadla	0.0119	(0.0114, 0.0124)	0.0000
443	Walisona Goro	0.0101	(0.0097, 0.0106)	0.0000
444	Walmara	0.0027	(0.0025, 0.0029)	0.0000
445	Wama Bonaya	0.0050	(0.0047, 0.0054)	0.0000
446	Wara Jarso	0.0148	(0.0143, 0.0155)	0.0000
447	Wegde	0.0202	(0.0193, 0.0211)	0.0000
448	Wegera	0.0136	(0.0130, 0.0142)	0.0000
449	Welkait	0.0093	(0.0088, 0.0100)	0.0000
450	Wembera	0.0049	(0.0045, 0.0054)	0.0000
451	Wenago	0.0021	(0.0019, 0.0024)	0.1180
452	Wenchi	0.0065	(0.0061, 0.0069)	0.0000
453	Were Ilu	0.0086	(0.0082, 0.0091)	0.0000
454	Werebabu	0.0084	(0.0080, 0.0089)	0.0000
455	WEREDA 06	0.0032	(0.0029, 0.0036)	0.0000
456	WEREDA 08	0.0030	(0.0027, 0.0033)	0.0000
457	WEREDA 12	0.0031	(0.0027, 0.0034)	0.0000
458	WEREDA 14	0.0033	(0.0030, 0.0037)	0.0000
459	WEREDA 16	0.0035	(0.0031, 0.0039)	0.0000
460	WEREDA 17	0.0037	(0.0033, 0.0041)	0.0000
461	WEREDA 19	0.0040	(0.0036, 0.0044)	0.0000
462	WEREDA 23	0.0035	(0.0032, 0.0039)	0.0000
463	WEREDA 24	0.0032	(0.0029, 0.0035)	0.0000
464	WEREDA 26	0.0045	(0.0040, 0.0049)	0.0000
465	WEREDA 27	0.0044	(0.0039, 0.0049)	0.0000
466	WEREDA 28	0.0034	(0.0030, 0.0037)	0.0000
467	Weremo Wajetuna Mida	0.0272	(0.0263, 0.0282)	0.0000
468	Werie Lehe	0.0174	(0.0166, 0.0182)	0.0000
469	Wuchalena Jido	0.0041	(0.0038, 0.0043)	0.0000
470	Wukro	0.0172	(0.0163, 0.0181)	0.0000
471	Yabelo	0.0054	(0.0049, 0.0058)	0.0000
472	Yalo	0.0087	(0.0082, 0.0094)	0.0000
473	Yaso	0.0068	(0.0064, 0.0073)	0.0000
474	Yaya Gulelena Debre Libanos	0.0051	(0.0048, 0.0054)	0.0000
475	Yayu	0.0028	(0.0027, 0.0030)	0.0000
476	Yeki	0.0054	(0.0049, 0.0059)	0.0000
477	Yem	0.0119	(0.0112, 0.0125)	0.0000
478	Yirgachefe	0.0027	(0.0025, 0.0030)	0.0000
479	Yobdo	0.0035	(0.0031, 0.0039)	0.0000
480	Zala	0.0054	(0.0050, 0.0058)	0.0000
481	Zikuala	0.0159	(0.0151, 0.0167)	0.0000
482	Ziway Gugda	0.0062	(0.0056, 0.0068)	0.0000
