# Supplementary material for: Model-based geostatistics enables more precise estimates of neglected tropical-disease prevalence in elimination settings: mapping trachoma prevalence in Ethiopia
Source: Int J Epidemiol. 2021 Nov 13;51(2):468–78. doi: 10.1093/ije/dyab227 (PMC9082807; doi:10.1093/ije/dyab227)
Supplement: dyab227_Supplementary_Data [file dyab227_supplementary_data.zip › ije-2020-07-1333-File010.pdf]

Supplementary Table S2: Details of the comparison of the geostatistical to the traditional analysis showing differences in prevalence and ratio of the 95% predictive interval length.

	Evalution Unit	Diff in Prevalence.	Ratio of 95% Predictive Interval Length
1	Abichuna Gne'a	0.0007	14.0525
2	Abobo	0.0093	12.1757
3	Achefer	0.0077	9.6310
4	Adami Tulu Jido Kombolcha	0.0043	8.7974
5	Adda Berga	0.0094	68.0010
6	Addi Arkay	0.0070	7.7792
7	Adwa	0.0097	7.2093
8	Ahferom	0.0028	8.0064
9	Akaki	0.0008	16.5049
10	Akililna Mohr	0.0012	9.5087
11	Akobo	0.0139	8.9410
12	Alaba	0.0058	10.5644
13	Ale	0.0010	5.8133
14	Alefa	0.0036	6.9370
15	Alem Gena	0.0049	11.1137
16	Aleta Wendo	0.0012	10.6540
17	Amba Sel	0.0048	9.9516
18	Ambo	0.0019	9.0670
19	Ameya	0.0005	21.2915
20	Angacha	0.0055	9.3049
21	Angolela Tera	0.0018	11.0876
22	Ankesha	0.0002	17.6018
23	Ankober	0.0109	10.6056
24	Antsokia Gemza	0.0081	12.2201
25	Arbe Gona	0.0005	10.0077
26	Aroresa	0.0008	9.6808
27	Artuma Fursina	0.0024	7.0543
28	Asagirt	0.0053	7.9677
29	Asegede Tsimbela	0.0117	9.7391
30	Atsbi Wenberta	0.0002	7.3786
31	Awabel	0.0090	7.5600
32	Awasa	0.0034	62.6007
33	Badawacho	0.0064	16.4380
34	Bako Gazer	0.0003	7.7898
35	Banja	0.0059	9.0182
36	Basketo	0.0029	7.4141
37	Baso Liben	0.0253	9.0400
38	Bati	0.0012	4.7330
39	Bedele	0.0011	16.8418
40	Belesa	0.0013	17.7332

	Evalution Unit	Diff in Prevalence.	Ratio of 95% Predictive Interval Length
41	Bensa	0.0016	14.4425
42	Berehna Aleltu	0.0015	9.6336
43	Bero	0.0086	11.6434
44	Beyeda	0.0001	7.2622
45	Bibugn	0.0406	44.9379
46	Bila Seyo	0.0026	9.9290
47	Boloso Sore	0.0118	9.0736
48	Bore	0.0017	123.1926
49	Borecha	0.0081	13.3887
50	Boreda	0.0040	11.1986
51	Boset	0.0133	38.2844
52	Bugna	0.0101	6.3174
53	Bulen	0.0018	8.1543
54	Bure	0.0000	8.2308
55	Bure Wemberma	0.0057	15.7049
56	Cheha	0.0122	12.8456
57	Cheliya	0.0012	15.5294
58	Chencha	0.0031	11.9081
59	Chilga	0.0115	35.2020
60	Chora	0.0054	12.4217
61	Dale	0.0013	17.2503
62	Damot Gale	0.0138	9.3582
63	Damot Weyde	0.0045	8.6153
64	Dangila	0.0032	7.0310
65	Dangur	0.0134	21.9295
66	Dano	0.0043	89.2828
67	Dara	0.0013	5.2054
68	Daramalo	0.0048	10.2700
69	Darimu	0.0013	11.4536
70	Dawa Chefa	0.0047	7.1213
71	Dawo	0.0147	90.2604
72	Dawunt Delanta	0.0017	9.0348
73	Debarq	0.0057	7.6728
74	Debay Telatgen	0.0234	54.9543
75	Debre Berhan Zuria	0.0022	7.3025
76	Debre Marqos	0.0365	28.1934
77	Debre Tabor	0.0015	6.4320
78	Dedesa	0.0027	27.4065
79	Dega	0.0025	10.5311
80	Dega Damot	0.0046	9.6445

	Evalution Unit	Diff in Prevalence.	Ratio of 95% Predictive Interval Length
81	Degem	0.0004	8.2712
82	Dejen	0.0190	9.0150
83	Dembia	0.0055	7.5871
84	Dendi	0.0002	7.5568
85	Dera	0.0036	10.4699
86	Dessie Zuria	0.0039	6.1354
87	Dibate	0.0054	12.3844
88	Diga	0.0009	8.3575
89	Dirashe	0.0034	7.3183
90	Dire Dawa	0.0008	16.6274
91	Dita	0.0048	12.6324
92	Efratana Gidim	0.0091	10.2547
93	Ejere (Addis Alem)	0.0029	144.4066
94	Enarj Enawga	0.0155	11.6905
95	Enbise Sar Midir	0.0150	7.4680
96	Endagagn	0.0014	13.2217
97	Enemay	0.0146	9.2921
98	Enemorina Eaner	0.0124	10.3131
99	Erob	0.0020	11.4972
100	Esite	0.0102	7.2823
101	Ezha	0.0157	14.9293
102	Fagta Lakoma	0.0077	11.4907
103	Farta	0.0005	34.3625
104	Fogera	0.0097	14.0826
105	Gambela	0.0125	17.1503
106	Ganta Afeshum	0.0070	9.7744
107	Gechi	0.0035	9.2370
108	Gelila	0.0009	7.3792
109	Gera Midirna Keya Gabriel	0.0061	9.7832
110	Gerar Jarso	0.0050	12.0240
111	Gidan	0.0071	11.4017
112	Gimbichu	0.0010	17.9352
113	Ginde Beret	0.0029	6.5395
114	Girawa	0.0160	39.2507
115	Gishe Rabel	0.0013	7.4454
116	Godere	0.0030	9.0004
117	Gofa Zuria	0.0198	73.2138
118	Gog	0.0082	8.1529
119	Goncha Siso Enese	0.1048	76.0007
120	Gonder Zuria	0.0039	6.1452

	Evalution Unit	Diff in Prevalence.	Ratio of 95% Predictive Interval Length
121	Goro	0.0161	11.4840
122	Guangua	0.0035	7.2811
123	Guba	0.0046	5.3993
124	Guba Lafto	0.0014	7.4656
125	Gulomahda	0.0065	7.9359
126	Guto Wayu	0.0051	7.9699
127	Guzamn	0.0016	6.1366
128	Hagere Mariamna Kesem	0.0053	23.7047
129	Harar/Hundene	0.0077	30.5539
130	Haro Maya	0.0022	10.3523
131	Hawzen	0.0073	8.2289
132	Hidabu Abote	0.0025	23.5531
133	Hulet Ej Enese	0.0064	6.5842
134	Hulla	0.0004	13.7447
135	Humbo	0.0060	11.3985
136	Ilu	0.0048	22.8920
137	Itang	0.0264	20.7116
138	Jabi Tehnan	0.0108	8.5554
139	Jama	0.0092	9.5506
140	Janamora	0.0102	31.3451
141	Jeldu	0.0028	12.2820
142	Jikawo	0.0145	8.4504
143	Jile Timuga	0.0031	9.9320
144	Jimma Arjo	0.0035	10.2192
145	Jor	0.0108	9.6087
146	Kacha Bira	0.0086	11.1377
147	Kafta Humera	0.0064	9.9602
148	Kalu	0.0032	8.7792
149	Kedida Gamela	0.0028	8.8530
150	Kelela	0.0085	9.3711
151	Kemba	0.0022	8.7900
152	Kembibit	0.0016	7.3399
153	Kersana Kondaltiti	0.0179	78.6052
154	Kewet	0.0080	7.3382
155	Kindo Koysha	0.0051	8.7824
156	Kobo	0.0041	6.8659
157	Kokir Gedbano Gutazer	0.0007	14.3511
158	Kokosa	0.0018	73.2569
159	Kola Temben	0.0145	8.2925
160	Kombolcha	0.0016	7.3675

	Evalution Unit	Diff in Prevalence.	Ratio of 95% Predictive Interval Length
161	Komesha	0.0075	14.5138
162	Konso	0.0036	17.1989
163	Kucha	0.0063	52.1673
164	Kurfa Chele	0.0049	7.9577
165	Kurmuk	0.0050	5.9848
166	Kutaber	0.0100	19.2810
167	Kuyu	0.0050	10.3126
168	Laelay Adiyabo	0.0078	7.7170
169	Lanfero	0.0234	45.1288
170	Lay Armacheho	0.0013	6.3062
171	Lay Betna Tach Bet	0.0098	8.1743
172	Leka Dulcha	0.0170	14.1227
173	Limu Kosa	0.0004	7.1467
174	Limu Seka	0.0014	12.7344
175	Lome	0.0037	12.2685
176	Machakel	0.0093	9.4463
177	Mama Midirna Lalo	0.0030	8.8514
178	Mandura	0.0079	17.6759
179	Masha	0.0027	94.7873
180	Medebay Zana	0.0046	8.4960
181	Mekdela	0.0083	9.4345
182	Meket	0.0059	7.5828
183	Menge	0.0056	10.3776
184	Merawi	0.0059	8.7468
